# Lessons Learned From Clinicians and Stroke Survivors About Using Telerehabilitation Combined With Exergames: Multiple Case Study

**DOI:** 10.2196/31305

**Published:** 2022-09-15

**Authors:** Dorra Rakia Allegue, Shane Norman Sweet, Johanne Higgins, Philippe S Archambault, Francois Michaud, William C Miller, Michel Tousignant, Dahlia Kairy

**Affiliations:** 1 The Centre for Interdisciplinary Research in Rehabilitation of Greater Montreal Institut universitaire sur la réadaptation en déficience physique de Montréal Montreal, QC Canada; 2 School of Rehabilitation Université de Montréal Montreal, QC Canada; 3 Department of Kinesiology and Physical Education McGill University Montreal, QC Canada; 4 School of Physical and Occupational Therapy McGill University Montreal, QC Canada; 5 Department of Electrical Engineering and Computer Engineering Université de Sherbrooke Sherbrooke, QC Canada; 6 Department of Occupational Science & Occupational Therapy University of British Columbia Vancouver, BC Canada; 7 School of Rehabilitation Université de Sherbrooke Sherbrooke, QC Canada; 8 Research Centre on Aging (CdRV) Université de Sherbrooke Sherbrooke, QC Canada

**Keywords:** stroke, rehabilitation, virtual reality, video games, telerehabilitation, upper extremity, motivation

## Abstract

**Background:**

In Canada, stroke survivors have difficulty accessing community-based rehabilitation services because of a lack of resources. VirTele, a personalized remote rehabilitation program combining virtual reality exergames and telerehabilitation, was developed to provide stroke survivors an opportunity to pursue rehabilitation of their chronic upper extremity (UE) deficits at home while receiving ongoing follow-up from a clinician.

**Objective:**

We aimed to identify the behavioral and motivational techniques used by clinicians during the VirTele intervention, explore the indicators of empowerment among stroke survivors, and investigate the determinants of VirTele use among stroke survivors and clinicians.

**Methods:**

This multiple case study involved 3 stroke survivors with chronic UE deficits and their respective clinicians (physiotherapists) who participated in the VirTele intervention, a 2-month remote rehabilitation intervention that uses nonimmersive virtual reality exergames and telerehabilitation aimed at improving UE deficits in stroke survivors. Study participants had autonomous access to Jintronix exergames and were asked to use them for 30 minutes, 5 times a week. The VirTele intervention included 1-hour videoconference sessions with a clinician 1 to 3 times a week, during which the clinician engaged in motivational interviewing, supervised the stroke survivors’ use of the exergames, and monitored their use of the affected UE through activities of daily living. Semidirected interviews were conducted with the clinicians and stroke survivors 4 to 5 weeks after the end of the VirTele intervention. All interviews were audiorecorded and transcribed verbatim. An abductive thematic analysis was conducted to generate new ideas through a dynamic interaction between data and theory.

**Results:**

Three stroke survivors (n=2, 67%, women and n=1, 33%, man), with a mean age of 58.8 (SD 19.4) years, and 2 physiotherapists participated in the study. Five major determinants of VirTele use emerged from the qualitative analyses, namely technology performance (usefulness and perception of exergames), effort (ease of use), family support (encouragement), facilitators (considerations of the stroke survivors’ safety as well as trust and understanding of instructions), and challenges (miscommunication and exergame limits). During the VirTele intervention, both clinicians used motivational and behavioral techniques to support autonomy, competence, and connectivity. All these attributes were reflected as empowerment indicators in the stroke survivors. Lessons learned from using telerehabilitation combined with exergames are provided, which will be relevant to other researchers and contexts.

**Conclusions:**

This multiple case study provides a first glimpse into the impact that motivational interviewing can have on adherence to exergames and changes in behavior in the use of the affected UE in stroke survivors. Lessons learned regarding the supportive role caregivers play and the new responsibilities clinicians have when using the VirTele intervention may inform the use of exergames via telerehabilitation. These lessons will also serve as a model to guide the implementation of similar interventions.

**International Registered Report Identifier (IRRID):**

RR2-10.2196/14629

## Introduction

### Background

In Canada, stroke survivors have difficulty accessing community-based rehabilitation services because of a lack of resources [1]. Evidence indicates that there is potential for recovery, even several years after stroke [1,2]. However, rehabilitation services are generally provided in the acute and postacute stages [1]. A common long-term consequence of stroke is hemiparesis, or weakness on one side of the body, leading to loss of upper extremity (UE) motor function with a significant long-term impact on everyday activities [3]. Given the chronic nature of stroke, it is essential to develop interventions that provide community-dwelling stroke survivors opportunities for further personalized training.

Telerehabilitation and virtual reality technologies could play an important role in providing novel rehabilitation approaches to optimize stroke recovery in the chronic phases, as suggested by Canadian stroke guidelines [1]. More specifically, telerehabilitation can be used to increase accessibility to rehabilitation programs and follow-up for persons no longer receiving rehabilitation (or discharged from intensive rehabilitation), whereas virtual reality technologies, which involve engaging activities for practice, can provide the intensity needed for optimal recovery. Moreover, behavioral and motivational techniques [4] could be used with these technologies to empower stroke survivors to continue exercising and using their affected UE in everyday activities (eg, brushing their hair, getting dressed, and eating). A few studies have examined the combined use of telerehabilitation and virtual reality technologies in stroke survivors [5-7]. These studies reported an improvement in UE motor function and high adherence to the treatment plan, which suggests that adding a motivational component to the technology may foster gains and changes in behavior in the long term.

### VirTele: Virtual Reality Combined With Telerehabilitation

VirTele, a personalized remote rehabilitation program combining virtual reality exergames and telerehabilitation, was developed to provide stroke survivors an opportunity to pursue rehabilitation of their chronic UE deficits at home while receiving ongoing follow-up from a clinician [8]. More specifically, VirTele used Jintronix exergames [9] and the Reacts platform (Koninklijke Philips NV) [10] to provide personalized training for the UE and enable videoconference sessions with a clinician, respectively. At the time of the intervention, the Jintronix exergames included 5 types of UE games (*Space Race*, *Fish Frenzy*, *Pop Clap*, *Apple Picking*, and *Kitchen Cleanup*) performed in a sitting position. The performance of the affected UE (score, percentage of compensation, and number of repetitions) and the duration and number of sessions played can be accessed on the web through a clinician portal within the Jintronix system. Reacts is an internet-based audiovisual platform that can be used through a computer or mobile phone to conduct secure videoconferences and share content (images, videos, messages, etc). It enables screen sharing (viewing the participant’s computer screen) to supervise in real time the stroke survivors’ performance, provide direct feedback, and adjust difficulty level in collaboration with the stroke survivors, taking into account their preferences and capacities (as observed during real-time Reacts sessions and through the data available on the Jintronix web portal).

An initial study was conducted with a stroke survivor to test the VirTele technology and study protocol during the development phase of the intervention [11]. The results showed that it was feasible to use the VirTele program for remote UE rehabilitation [11]. Meaningful determinants of technology use were identified, including performance (perceived improvement in UE use during daily activities and unlimited time of exercises), effort (feeling comfortable using VirTele and experiencing only minor technical issues, which the stroke survivor could easily resolve), and social influence (positive feedback from family and friends) [11]. Preliminary efficacy results showed improvement in UE motor function, UE quality and quantity of use in activities of daily living, and quality of life [11]. Hence, there is interest in studying this technology further to explore varied experiences among more participants, including clinicians and stroke survivors.

### Sustaining Gains Through Behavior Modification and Shared Decision-making

Behavior-modification strategies (eg, patient-centered counseling, action planning, and self-monitoring) have been implemented in exercise promotion interventions to enhance motivation, exercise participation, and maintenance [12-14]. As gains achieved during rehabilitation are not always maintained in the long term [15], chronic-stroke survivors may benefit from such behavior-modification strategies when they are integrated into postrehabilitation programs. These strategies could be used to empower stroke survivors to continue exercising and using their UE in everyday activities (eg, brushing their hair, getting dressed, and eating). Furthermore, there is increased recognition that programs aimed at changing behaviors should have a strong theoretical basis [16]. Self-determination theory (SDT) [17] states that human beings have a natural tendency to autonomously pursue goals or achieve healthy changes when 3 of their psychological needs are satisfied, namely autonomy (a person’s ability to act according to their own values and aspirations), competence (a person’s belief in their ability to achieve changes), and connectivity (a feeling of belonging) [17]. Therefore, social environments where the clinician engages in a partner relationship with the stroke survivor while supporting their autonomy (shared decision-making, choice of exergames, etc), competence (the stroke survivor’s belief in their capacity to achieve their goals, etc), and connectivity (a nonjudgmental interaction) may result in greater autonomous motivation [18]. Previous studies [19,20] demonstrated that support of the 3 psychological needs predicted greater autonomous motivation, which resulted in better adherence to exercises. A recent meta-analysis of SDT-informed interventions [21] found small-to-medium effects of physical health outcomes (physical fitness and function, weight-related outcomes, blood pressure, etc) at the end of the interventions and during the follow-up period (ranging from 1 week to 30 months after the interventions). As autonomous motivation is a key element for developing maintained change, a supportive psychological needs environment should be integrated into the VirTele intervention. Thus, motivational interviewing [22], consistent with SDT, was incorporated into the VirTele program to ensure that shared decision-making and empowerment were consistently integrated into the intervention. The behavioral and motivational techniques incorporated into motivational interviewing may enhance autonomous motivation to adhere to the treatment plan and change behavior regarding UE use in activities of daily living. In addition, combining real-time videoconferencing (telerehabilitation) with virtual reality technology could allow for adequately monitored and engaging theory-based UE rehabilitation programs, which may enhance stroke survivors’ empowerment and sustain gains in the long term.

Eventually, the SDT-informed VirTele intervention may not only help patients and clinicians decide together on the best treatment options but also allow clinicians to identify potential problems once the patient has reintegrated into the community. Thus, this study will also document the experiences of the stroke survivor as well as the clinician when using the VirTele program, which are key aspects for the successful eventual implementation of such interventions.

The objectives of this study were as follows:

1. Identify behavioral and motivational techniques used by clinicians during the VirTele intervention.
2. Explore indicators of empowerment among stroke survivors.
3. Investigate the determinants of VirTele use among stroke survivors and clinicians.

## Methods

### Study Design

This study used a multiple case design, which allows extensive data collection with varied methods across different cases [23]. This design enables the exploration of the studied phenomenon across a more varied range of characteristics compared with a single-case model [23]. The unit of analysis in this multiple case study is each stroke survivor and their respective clinician (physiotherapist) participating in the VirTele intervention. A range of experiences in terms of age, sex, familiarity with technology, and living arrangements were sought.

### Context

This multiple case study is embedded into a 2-armed randomized clinical trial comparing an experimental group (receiving the VirTele intervention) with a control group (receiving standard care) in Montreal, Quebec, Canada, and registered with ClinicalTrials.gov (NCT03759106) [8].

The qualitative data were collected between June 2019 and August 2020 by the first author (DRA; who was not involved in the VirTele intervention), a PhD student under the supervision of DK and JH who had previous experience in qualitative research and stroke rehabilitation research. This multiple case study was reported according to the Standards for Reporting Qualitative Research [24].

### Sampling Strategy and Participants

This study targeted the stroke survivors who were assigned to the experimental group receiving the VirTele intervention in the context of the 2-armed randomized clinical trial and who had completed the 2-month program. This group of stroke survivors was screened for eligibility before enrollment, and participants were selected based on the inclusion and exclusion criteria described in the published protocol (refer to the Participant Selection and Recruitment Strategy section) [8]. The clinicians included in the main study were physiotherapists who had experience with stroke rehabilitation. All participants had to speak French or English.

### Ethical Considerations

This study received ethics approval from the research ethics board of the Centre for Interdisciplinary Research in Rehabilitation of Greater Montreal (review number CRIR-1319-0218; June 28, 2018) [8]. All participants provided informed written consent before starting the VirTele intervention.

### VirTele Protocol

**Figure 1:**
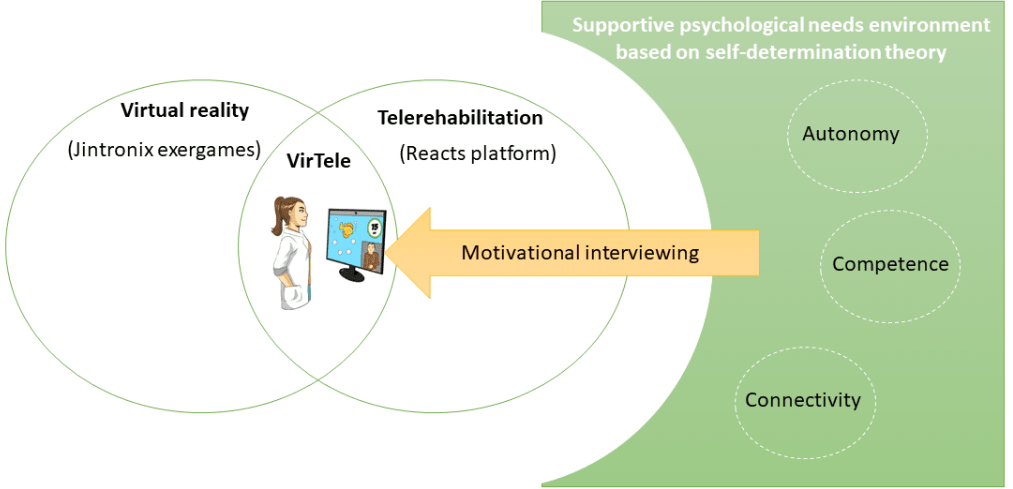
Representation of the theoretical and technological components of the VirTele intervention.

The VirTele intervention protocol is illustrated in Figure 1. The stroke survivors were invited to use Jintronix exergames for 30 minutes at least 5 times a week and conduct 1-hour videoconference sessions with a clinician, using Reacts, for a period of 2-months. The videoconference sessions took place 3 times a week for the first 2 weeks, twice a week for the next 2 weeks, and then once a week for the remaining 4 weeks [8]. The clinicians only received training to familiarize themselves with the VirTele technology (Jintronix exergames and Reacts platform) because they were already trained in motivational interviewing (including SDT concepts), an approach they had been using in their practice for >2 years before the study began. Although SDT and motivational interviewing were developed independently, a resemblance exists between them [25]. In fact, motivational interviewing techniques [4] are consistent with the 3 psychological needs of SDT [25]; for example, motivational interviewing promotes shared decision-making (selection of treatment goals, exercises, etc), behavior change techniques (such as express advantages and disadvantages of change, goal setting, and review of goals) [26], and reflective listening (shows empathy), which emphasize autonomy, competence, and connectivity, respectively [4]. Thus, motivational interviewing, including motivational and behavioral techniques, was achieved through videoconferencing sessions conducted using the Reacts platform (Figure 1).

### Determinants of VirTele Acceptability Among End Users

The Unified Theory of Acceptance and Use of Technology (UTAUT) [27] was used to explore the factors that may influence VirTele adoption intention and use behavior among stroke survivors and clinicians, to fulfill the third objective of this study. The UTAUT states that the intention to adopt a new technology is determined by 3 main factors: expected performance (the degree to which the technology is perceived as helpful and useful), expected effort (ease of use and complexity associated with using the technology), and social influence (positive or negative feedback that family and friends may have regarding the technology) [27]. In addition to the intention to adopt a new technology, contextual conditions (such as the ability and knowledge to use a new technology and interoperability) may facilitate the use behavior regarding the technology [27].

The UTAUT also incorporates 4 moderators—age, sex, experience, and willingness to use—that can influence technology adoption intention and use behavior [27]. In the context of this study, it is interesting to capture the expectations of users as well as their actual experiences to see whether the technology meets the needs of end users. Operational definitions of the UTAUT and concrete examples are provided in Multimedia Appendix 1.

### Data Collection

Triangulation was used for this multiple case study. This involved the use of various methods to collect qualitative data [28]. First, semistructured interviews (lasting from 30 minutes to 1 hour) were conducted 4 to 5 weeks after the end of the VirTele intervention with the stroke survivors and clinicians. Two interview guides were developed and tailored to the clinicians (eg, What was your role or responsibility during VirTele? Did you have any concerns when you first started using the technology?) and the stroke survivors (eg, Did you perceive any change in your arm function? Can you describe this change?) to facilitate the interview administration while allowing new ideas to emerge. Questions were structured to target the key concepts of each theory. Key UTAUT concepts were used to identify major factors that influenced the VirTele experience in the stroke survivors as well as the clinicians. For the stroke survivors, SDT concepts were used to explore the indicators of empowerment in terms of autonomy, competence, and connectivity. For the clinicians, SDT was used to identify which motivational interviewing technique was used and which need was supported (autonomy, competence, or connectivity) when interacting with the stroke survivors. The interview took place either face to face at the research center or remotely through the Reacts platform.

Second, logbooks were used by the clinicians to collect data related to technical difficulties, number of videoconference meetings, complementary activities suggested in addition to the exergames, and motivational strategies used. Third, reflexive notes were used by the researchers to collect VirTele intervention–context-related data (technical difficulties, adverse events, etc). Demographic information about the stroke survivors was also collected. A sample size of 10 stroke survivors and 4 clinicians was targeted to diversify the experiences and enrich the data. However, only data saturation can predict the final sample size [29].

### Data Analysis and Processing

Abductive thematic analysis was conducted. This type of analysis seeks to go beyond inductive and deductive reasoning [30]. By adopting this approach, researchers can generate new ideas through a dynamic interaction between data and theory [30]. First, a predetermined coding scheme was developed based on UTAUT and SDT constructs. Next, the transcript text was examined to identify which meaning unit reflected one of the predetermined codes. Codes and assemblies were frequently revised, and relevant new codes were assigned to meaning units that could not be coded or categorized within the initial scheme codes. Finally, the new codes were examined and either represented as subcategories (reflecting border concepts related to the UTAUT or SDT) or new categories of codes (enriching the corpus of existing theories).

QDA Miner (Provalis Research) [31] was used to enter the list of predetermined scheme codes and retrieve the highlighted text into meaning units, which were condensed and then coded and categorized using the scheme codes.

In each case, the stroke survivors’ and clinicians’ experiences with the VirTele intervention and indicators of SDT variables were developed and examined independently and across the duos (stroke survivors and clinicians) for a within-case comparison. Next, experiences were examined among cases, using a cross-case analysis, to explore differences and similarities regarding the determinants of VirTele use and indicators of SDT variables. Underlying similarities and constant associations were then developed to form more general explanations. The analysis was conducted by 3 members of the research team (DRA, DK, and JH). The verbatim transcripts were translated from French into English for publication and verified by bilingual team members (DRA and DK).

### Rigor

The principles of Lincoln and Guba [32], including confirmability, credibility, reliability, and transferability, were applied to ensure study rigor. Audit trails and verification were conducted to ensure confirmability. An external verification by members was carried out for credibility. Reliability was confirmed through verification of a portion of the data by 3 coders (DRA, DK, and JH). For transferability, reflexive notes and a detailed description of the context of the intervention were compiled. The variation in the cases may increase the robustness of the qualitative data [29].

## Results

### Sociodemographic Data of Stroke Survivors

Five stroke survivors were assigned to the intervention group and completed the VirTele intervention (Table 1).

**Table 1:** Stroke survivor sociodemographic data.

Variable	Stroke survivor ID				
	1	2	5	10	11
Age (years)	41	67	89	47	50
Sex	Female	Male	Female	Male	Male
Dominance	Right-handed	Right-handed	Right-handed	Right-handed	Ambidextrous
Year of stroke	2014	2011	2014	2010	2017
Stroke side	Right	Left	Right	Right	Right
Chedoke-McMaster stroke assessment score	Stage 3	Stage 3	Stage 5	Stage 4	Stage 4
Living arrangement	Living with family	Living with spouse	Living with daughter	Living alone	Living with spouse
Computer familiarity	Very comfortable, accessible at home, and use less than once a month	Comfortable, accessible at home, and use one or more times a week	Not comfortable, accessible at home, and never use	A little comfortable, accessible at home, and use once a week	Very comfortable, accessible at home, and use one or more times a week

However, recruitment was halted in mid-March 2020 at the onset of the COVID-19 pandemic in Canada, and all research activities were suspended from March 2020 to October 2020. Of the 5 stroke survivors allocated to the VirTele group, 1 (20%) could not be reached to conduct the interview, and 1 (20%) was excluded because he did not speak French or English fluently. Of the 3 remaining stroke survivors, 2 (67%) were women (participant ID1 and participant ID5), and 1 (33%) was a man (participant ID11); their mean age was 58.8 (SD 19.4) years, and they varied in terms of computer familiarity, Chedoke-McMaster stroke assessment score, time since stroke, and dominance of UE. Two physiotherapists participated in administering the VirTele intervention. Participant ID11 received a 3-month VirTele intervention instead of 2 months, as was the case for participant ID1 and participant ID5, given that it was impossible to retrieve the technology material during the COVID-19 pandemic period. We decided to give this participant the opportunity to benefit from the services offered by this technology for an additional month. For readability, each participant was given a pseudonym: participant ID1 identified as Carolina, participant ID5 identified as Helene, and participant ID11 identified as Jack.

### Case Description and Comparison

A detailed case description of the 3 duos (stroke survivor and respective clinician), collected from the interviews, logbooks, and exergame portal, is provided in Multimedia Appendix 2. A summary of the techniques used by the clinicians during motivational interviewing and their impact on stroke survivor empowerment, collected from logbooks and interviews, is provided in Table 2. The differences among the 3 cases are illustrated in Multimedia Appendix 3. The determinants of VirTele use, as expressed by the stroke survivors and clinicians during the interviews, are presented in Table 3. Although we did not reach our target sample size because of the COVID-19 pandemic, the data collected from the 5 participants allowed us to achieve a certain level of data saturation because many of the reported experiences were repeated across cases.

**Table 2:** Indicators of support of psychological needs and empowerment^a^.

Category	Support of psychological needs by the clinician		Stroke survivor empowerment
	Technique used to change behavior^b^	Strategies specific to VirTele^c^	
**Autonomy**			
	9.2: Allows participant to express advantages and disadvantages	Gives the participant an opportunity to talk about UE^d^ use in daily activities and the difficulties encountered	Speaks about UE use in daily activities
	N/A^e^	Changes the difficulty parameters of the exergames according to participant preferences	Chooses the parameters of difficulty in exergames (“Make it faster, make it slower”)
	N/A	Shared decision-making	Makes decisions related to choice of exergames and level of difficulty
**Competence**			
	15.1: Verbal persuasion about capability	Answers participants’ questions and helps solve problem discussed	“If I had a problem or a question, I’d text him”
	1.1: Goal setting	Shows the participant how to perform stretches and exercises with affected arm	Feeling supported to perform exercises and arm stretches through demonstration and encouragement
	1.5: Review of goals	Demonstrates exercises in exergames	Feels supported to play exergames and use UE in activities of daily living because of advice given on performance
	1.1: Goal setting	Gives advices on performance during exergames	Feels supported to use exergames because of advice, demonstrations, and feedback
	1.4: Action planning	Celebrates small successes	N/A
	1.2: Problem-solving	Encourages participant to maintain some postures, even for a few seconds	N/A
	2.7: Feedback on behavior results (positive feedback)	N/A	N/A
	2.2: Feedback on behavior	N/A	N/A
	7.1: Prompts and cues	N/A	N/A
**Connectivity**			
	N/A	Has a calm way of speaking	Feels comfortable and finds it easy to be around, and work with, the clinician
	N/A	Establishes a trust relationship	Feels comfortable interacting with the clinician
	N/A	Uses reflective listening (expresses empathy)	Finds the clinician to be kind
	N/A	Listens and acknowledges the participant’s opinion	N/A
	N/A	Is patient and enthusiastic	N/A

^a^The indicators of support of psychological needs and empowerment for each participant are provided in more detail in Multimedia Appendix 3 to reflect the differences and similarities among the 3 cases.

^b^The behavior change techniques reported in the table are based on the taxonomy of Michie et al [26], who proposed 93 clustered behavior change techniques. To make it easier for the reader to find the techniques used in our study in the taxonomy of Michie et al [26], the number assigned to each technique is reported in the table.

^c^A program that combines nonimmersive virtual reality exergames and telerehabilitation.

^d^UE: upper extremity.

^e^N/A: not applicable.

**Table 3:** Determinants of VirTele^a^ use.

Category	Subcategory of codes	
	Stroke survivors	Clinicians
**Performance**		
	Relative advantage	Relative advantage
	Perceived limits of exergames	Stroke survivor empowerment
	Stroke survivors’ perception of exergames	Perceived limits of exergames
	Perceived change in the affected arm use	Stroke survivors’ perception of exergames
	Awareness of usefulness of the technology	Perceived change in the affected arm use
	Stroke survivors’ adherence to exergames	Clinician’s instructions and demonstrations of exercises through technology
	Stroke survivors’ experience with exergames	Clinicians are apprehensive about demonstrating exercise through technology
	N/A^b^	Clinicians’ role in VirTele context
**Effort**		
	Managing technical issues	Managing technical issues
	Perceived ease of use of the technology	Perceived ease of use of the technology
**Social influence**		
	Feedback from family and friends, agreement, and assistance with the technology	N/A
	Clinician support and encouragement	N/A
**Contextual facilitating conditions**		
	N/A	Stroke survivor safety
	N/A	Stroke survivor capacity to understand the clinician’s instructions
	N/A	Trust between clinicians and stroke survivors
	N/A	Clinicians’ apprehension related to stroke survivors’ trust
**Contextual challenges**		
	Comfort in using the technology	Miscommunication between the stroke survivor and clinician
	Internet access	N/A
	Miscommunication between the stroke survivor and clinician	N/A

^a^A program that combines nonimmersive virtual reality exergames and telerehabilitation.

^b^N/A: not applicable.

### Determinants of VirTele Use

Differences and similarities have emerged regarding the determinants of VirTele use between the duos (stroke survivor and clinician).

#### Performance

##### Relative Advantages

In terms of relative advantages, the clinicians believed that VirTele facilitated access to rehabilitation services and that exergames and follow-up enhanced stroke survivor motivation and compliance to the rehabilitation program. A clinician felt that the feedback (scores of games and clinician feedback) and the follow-up increased stroke survivor empowerment. Neither the clinicians nor the stroke survivors expressed expectations regarding the benefits of the VirTele program, and only apprehensions were reported.

##### Stroke Survivors’ Perceptions of Exergames

The stroke survivors had different perceptions of the exergames (perceived either as an instrument of play or a therapeutic intervention). Helene compared the exergames with “bridge card games” and stated that she liked to win, which motivated her to continue playing during the 2-month intervention. Jack initially showed some apprehension, which diminished with practice, regarding the therapeutic value of the exergames.

##### Perceived Change in Use of Affected Arm

All stroke survivors demonstrated high adherence to the exergames; however, only Carolina and Helene expressed an intention to use the affected UE in daily activities, which was maintained after the end of the VirTele intervention. Jack had expressed no intention to use the affected UE in daily activities, which was corroborated by the clinicians. Helene experienced no improvement in motor function. She reported no change in her arm function but said that she had begun to use her arm in daily activities.

##### Clinicians’ Role in VirTele Context

From the clinicians’ perspective, their main role when using VirTele can be summarized in terms of the following tasks: adjust the difficulty level of the exergames, monitor the stroke survivors’ adherence to the exergames and their compliance to carrying out activities of daily living, observe the movements during the exergames, correct postures and movements, and act as coaches to motivate the stroke survivors and encourage and maintain adherence.

##### Instructions and Demonstration of Exercises Through Technology

With regard to demonstrating the exercises through the videoconference technology, without physical contact (hands-on demonstrations), the clinicians reported considerable apprehension, which subsided later because the stroke survivors were able to correctly comprehend the instructions. In addition, the clinicians were able to demonstrate the exercise through clear, concise, and simple instructions, which was challenging at times because of the participants’ loss of attention (not listening to the instructions or sound getting cut off).

##### Perceived Limits of Exergames and Stroke Survivors’ Experience

The clinicians pointed out some limits of the exergames that may influence technology performance, such as limited choice of exergames, which could become repetitive (significant focus on shoulder movements); limited parameters of difficulty; and insufficient rest time between sets of repetitions (users need to click the pause button manually). This feedback was provided by Jack. According to one of the clinicians, the lack of diversification in the difficulty parameters may induce a ceiling effect in terms of difficulty, which can be demotivating for the stroke survivor.

Helene and Carolina reported a problem with the avatar in some of the games (the avatar did not always follow the real movements). The clinicians believe that the avatar issues were related to not following recalibration instructions before starting the game, an important phase that allows the Kinect camera (Microsoft Corporation) to capture both arms and recalibrate the degrees of movement in each limb, enabling better control over the avatar.

#### Effort

With regard to effort, the clinicians as well as the stroke survivors encountered technological issues (eg, the screen froze or slowed down, and the sound or the internet connection were cut off), which caused some frustration among the stroke survivors. The issues were managed either by the research team or the clinician (telephone support) or by the stroke survivors themselves or with the help of a family member (restarting the computer, reconnecting to the internet, etc).

All of the clinicians and stroke survivors, except Helene, found the technology intuitive and user friendly. Helene needed the help of a family member to turn on and use the VirTele intervention.

#### Social Influence (Only for Stroke Survivors)

Positive feedback from friends and family, after seeing or hearing about the system, encouraged the stroke survivors to start or continue using the VirTele intervention. The clinicians also played an important role in supporting (demonstration, instructions, advice, etc) and encouraging the stroke survivors to adhere to the exergames and use the affected UE in daily activities. This may have contributed to their empowerment. Further details regarding stroke survivor empowerment are provided in Table 2.

#### Contextual Facilitators and Challenges

According to the clinicians, 3 main factors facilitated their use of the VirTele intervention: the stroke survivors’ safety (the exergames were performed in a sitting position, and no adverse events occurred), the capacity of the participants to comprehend their instructions through the technology, and the trust relationship established with the stroke survivors (through shared decision-making), regarding which the clinicians were apprehensive before the intervention.

The main challenge encountered by a clinician with Jack was that the stroke survivor had been diagnosed with aphasia. This led to miscommunication between the clinician and Jack, as well as frustration for the latter. Thus, the clinician encountered difficulty in carrying out the motivational interviews and customizing the intervention according to Jack’s needs because these were not well understood. Furthermore, challenges related to lack of comfort in using the technology (unfamiliarity with computers) and limited access to the internet were problems that both Helene and Jack had to deal with.

### Clinicians’ Recommendations Regarding the Use of the VirTele Intervention

In the clinicians’ interviews, some meaning units, reflecting different recommendations related to the use of the VirTele program, were assembled. They are presented as a bulleted list in Multimedia Appendix 4. Lessons can be learned from these recommendations (Textbox 1) regarding the use of telerehabilitation combined with exergames. In fact, these lessons provide relevant instructions for the use of exergames via telerehabilitation and suggest useful strategies to optimize the potential of this technology for the rehabilitation of the affected UE.

**Lessons learned about using telerehabilitation combined with exergames.**

**Lessons learned from clinicians’ recommendations**

- Both stroke survivors and clinicians are receptive to using the technology, despite technological limitations.
- The caregiver has a supportive role in using the technology, particularly among stroke survivors who are not familiar with IT.
- The clinicians’ transition into the new roles and responsibilities may be facilitated by considerations of the stroke survivors’ safety, capacity to understand the instructions, and trust.
- Aphasia may lead to frustration among stroke survivors when interacting with clinicians, but it does not affect technology use.
- The use of telerehabilitation combined with exergames may empower stroke survivors, through autonomy, competence, and connectivity, and increase frequency of use of the affected upper extremity in activities of daily living during and after the end of the VirTele intervention.

## Discussion

The objectives of this multiple case study were to (1) identify behavioral and motivational techniques used by clinicians during the VirTele intervention, (2) explore indicators of empowerment among stroke survivors, and (3) investigate the determinants of VirTele use among stroke survivors and clinicians.

### Principal Findings

#### Indicators of Empowerment and Support of Psychological Needs

The clinicians used numerous motivational interviewing strategies that helped to create supportive psychological needs environments. The stroke survivors demonstrated empowerment at different levels in term of autonomy, competence, and connectivity. This is likely to result in better management of self-care, more independence from clinicians, and increased motivation to pursue a rehabilitation program [33]. In fact, all participants used the exergames and achieved a great amount of autonomous use of the platform (the number of autonomous exergame sessions ranged from 37 to 68). More importantly, Carolina and Helene continued using their affected UE in daily activities and self-directed exercises after the end of the VirTele intervention.

Jack did not express any intention to use his UE in daily activities after the end of the VirTele program, although he used his UE in self-directed exercises during the VirTele program as per the clinician’s recommendations and instructions. Jack may have been externally motivated, which means that he wanted to change only for external reasons, not because he wanted to; for example, he performed an exercise because the clinician asked him to, or he used the exergames because he knew that he was being monitored. In addition, Jack’s indicators of empowerment were less developed at the connectivity and competence levels, which can be explained by the miscommunication challenge that he faced (because of his aphasia diagnosis). In fact, Jack’s clinician pointed out that Jack’s needs were not well understood. This made it difficult to customize the program and provide adequate support for competence and left little space for a sense of connectivity and belonginess. Therefore, the lack of participant empowerment in terms of autonomy, competence, and connectivity may reflect externally regulated motivation, rather than internal motivation, which often involves short-term changes (eg, stopping use of the UE after the end of the VirTele program).

Helene also demonstrated external motivation because she stated that she continued to use the exergames to win, not to exercise her UE, because she did not perceive any significant change with her UE. However, external motivation can be internalized and accepted to lead to effective changes [17]. At the end of the VirTele intervention, Helene reported that she had started self-directed exercises to avoid deterioration of her health condition and even started using her affected UE more frequently, which may reflect a self-regulated or self-identified motivation [17]. It is also important to note that other factors may increase autonomous motivation in stroke survivors, such as enjoyment during exergames or when improvements are perceived. This should be further examined in future studies.

Furthermore, recommendations reported by the clinicians reflecting what they learned from using VirTele were also provided, although these data were supplementary to, and not the original focus of, the study (Multimedia Appendix 4). These recommendations can be relevant to other researchers and transferable to other populations and contexts when incorporating virtual reality and telerehabilitation technologies.

#### Determinants of VirTele Use

Among the main determinants that were identified from the UTAUT, performance stood out as being meaningful in the 3 cases. In fact, the clinicians as well as the stroke survivors perceived relative advantages of the VirTele intervention compared with standard therapy (facilitating access to therapy and enhancing motivation) and felt comfortable interacting with each other.

The main role of the clinician during the VirTele intervention was to monitor the use of the affected UE by the stroke survivor through self-directed exergames and activities of daily living, which aims to enhance the stroke survivor’s autonomy to continue using their affected UE after the end of the intervention. This is particularly relevant in the chronic stage of stroke because not all stroke survivors have access to rehabilitation services after discharge [1]. The VirTele program could be offered at the end of inpatient rehabilitation to learn how to self-manage the UE rehabilitation at home, while being closely monitored by a clinician.

The limits of the exergames, as pointed out by the clinicians and stroke survivors, may have reduced the technology performance with regard to attaining the individual stroke survivors’ goals. However, it did not seem to affect the behavioral intention and use behavior regarding the technology among the clinicians and stroke survivors. Furthermore, family members ended up playing a supportive role (managing technical difficulties, supporting technology use, and motivating the participant and encouraging VirTele use) during the VirTele program, particularly with Helene who was not familiar with computers.

Communication difficulties, such as those resulting from Jack’s aphasia diagnosis, were considered the main challenge to motivational interviewing administration, which led to frustration for Jack, but did not affect technology use among the stroke survivors. Furthermore, 3 factors were identified by the clinicians as facilitators of technology use including trust, considerations for the participants’ safety, and their capacity to comprehend the clinician’s instructions. In addition to these factors, the previous experiences of the clinicians in motivational interviewing and their ease of use of the VirTele intervention may have facilitated the transition to their new roles and responsibilities in the VirTele context. This also suggests that the VirTele intervention may be easily transferred into actual clinical practice to offer stroke survivors opportunities for practice and to change their unhealthy behaviors.

### Comparison With Prior Work

This study’s results corroborate the findings in the study by Caughlin et al [34], which confirmed the supportive role of caregivers during telerehabilitation interventions (facilitating the use of the technology). The high level of adherence to the exergames and the increased use of UE in stroke survivors echo the findings of a previous systematic review [21], which found that interventions involving tailored counseling strategies such as goal setting and monitoring, motivational interviewing, and follow-up seem to be effective at promoting long-term physical activity participation after stroke. Furthermore, the use of the affected UE in self-directed exercises may result in improved motor function. At this stage of the study only the evaluations of the first few participants of the randomized clinical trial [35] were performed, and firm conclusions cannot be drawn regarding the results obtained on the sensorimotor measures. However, a trend in improvement was observed regarding motor function measured using the Fugl-Meyer Assessment [35] in Carolina and Jack as well as UE activity measured using the Motor Activity Log [35] (quality and quantity of use) in all participants. These gains were maintained 2 months after completion of the VirTele intervention [35]. The high adherence to the exercise program demonstrated by the participants could optimize the motor gains. In addition, the change in behavior with respect to the use of the UE in daily activities, as observed in Carolina and Helene, could justify the maintenance of the gains in the long term. Furthermore, it is important to note that Jack, who did not intend to use the affected UE after the end of the VirTele program, still managed to maintain long-term gains (improvements noted in the Fugl-Meyer Assessment and Motor Activity Log scores [35]), highlighting the importance of adhering to the VirTele program and its potential to maximize gains.

In a previous study, Sit et al [36] found that stroke survivors (n=105) receiving motivational techniques similar to those in our study (encouragement, verbal persuasion, goal setting, partner relationship between the clinician and the patient, action plan, and self-management steps) significantly improved functional indices (Barthel and Lawton indices, which are scales used to assess activities of daily life performance on independent living) and self-management outcomes (medication adherence, self-monitoring of blood pressure, communication with physician, etc) compared with a control group receiving standard care. Hence, further research is needed to explore the correlation between motivational interviewing and UE motor function outcomes among stroke survivors.

Moreover, the determinants of VirTele use, identified through this study, are in part consistent with the determinants reported by other studies deploying telerehabilitation [37] and virtual reality exergames [38]. Despite the technical issues, the 3 participants were receptive to the VirTele program and continued using the system, which echoes the findings of previous studies among stroke patients and clinicians using telerehabilitation [34].

### Limitations

Participants’ expectations regarding the VirTele intervention before the start of the study were not documented. Therefore, it was not clear whether the intervention met the participants’ expectations, which could affect technology acceptability and their compliance to the rehabilitation program. Future studies could investigate expectations before the start of the intervention to better capture end user expectations of similar interventions. Furthermore, given the small sample size, the results of this study should be interpreted with caution.

### Conclusions

In conclusion, the factors predicting intention to use the VirTele intervention and use behavior among stroke survivors and clinicians include technology performance, effort, social influence, contextual facilitators, and challenges. The empowerment attained by stroke survivors is promising for the future deployment of such an intervention to encourage the use of the affected UE in activities of daily living and achieve impactful long-term improvement. The lessons learned from this study regarding the resilience of stroke survivors and adaptability of clinicians with respect to technology limitations, role of the caregiver, new responsibilities of clinicians during the VirTele intervention, impact of aphasia diagnosis, and empowerment of stroke survivors may help to guide the implementation of similar interventions. However, further studies in different contexts are needed to better understand the factors affecting intention to use such technologies and use behavior.

## Appendix

Multimedia Appendix 1 Operational definitions.

Multimedia Appendix 2 Case description.

Multimedia Appendix 3 Indicators of empowerment and support of psychological needs among the 3 cases.

Multimedia Appendix 4 Clinicians’ recommendations regarding using telerehabilitation combined with exergames.

## Abbreviations

SDT: self-determination theory
UE: upper extremity
UTAUT: Unified Theory of Acceptance and Use of Technology
