# Supplementary material for: Lessons Learned From Clinicians and Stroke Survivors About Using Telerehabilitation Combined With Exergames: Multiple Case Study
Source: JMIR Rehabil Assist Technol. 2022 Sep 15;9(3):e31305. doi: 10.2196/31305 (PMC9523527; doi:10.2196/31305)
Supplement: Multimedia Appendix 1 [file rehab_v9i3e31305_app1.pdf]

Multimedia Appendix 1. Operational definitions

Operational definitions of the concepts have been developed based on potential users (clinician and survivor) and are presented below, including concrete examples for a common understanding of the proposed conceptual framework.

Performance: the degree to which the user perceives the technology as efficient, in other words, useful and advantageous compared to standard practice. This definition includes both expected and actual performance, as it aims to capture the user's expectations of the technology and the actual experience of the technology, which might have an impact on the user's motivation to continue using the system (same principle applies for the rest of the determinants).

Clinicians: This could translate into ease of designing rehabilitation exercises (adapted levels of difficulty, control of the duration and number of repetitions, various exercises, etc), ability to follow up (video conference, secure sharing of documents, images, video), added value to standard practice (playfulness of games, motivation of the survivor, personalization of treatment) and functional results of the survivor.

Stroke survivors: This could be reflected in the ability of the technology to improve access to rehabilitation programs (follow-up by a clinician, access to a personalized exercise program, etc.), to facilitate the performance of exercises in terms of duration, frequency, level of difficulty, etc., and to achieve satisfactory results in the motor performance of the arm (eg, use of the arm in activities of daily living).

Effort: the degree of ease and / or complexity associated with using the technology.

Clinicians and stroke survivors: This could translate into the effort required to use technology including learning or training in the use of exergames and telerehabilitation platforms, ease of interaction with platforms (launch games, start a video conference call, etc), period of familiarization with the technology, management of technical problems (the Internet, system update, forgotten passwords, etc), redefinition of the role and responsibilities.

Social influence: the degree of impact that positive or negative feedback from those around you can have on the use of Virtual Reality - Telerehabilitation technology.

Clinicians: Influence of co-workers, director, manager, etc.

Stroke survivors: Influence of family members, friends, neighbors, etc.

Facilitating conditions and obstacles: factors that can facilitate or inhibit the use of the technology including internal (ability and knowledge to use a new technology) and external (technical and organizational infrastructure) constraints or the degree of compatibility of the technology with user needs.

Clinicians: interoperability of systems, knowledge and ability to use the system, access to a computer, access to the Internet, compatibility with clinical reasoning, participant safety

Stroke survivors: knowledge and ability to use the system, access to adequate space (enough space to position themselves well in front of the Kinect camera and move their arm), participant safety during exercises.
