# Supplementary material for: Lessons Learned From Clinicians and Stroke Survivors About Using Telerehabilitation Combined With Exergames: Multiple Case Study
Source: JMIR Rehabil Assist Technol. 2022 Sep 15;9(3):e31305. doi: 10.2196/31305 (PMC9523527; doi:10.2196/31305)
Supplement: Multimedia Appendix 2 [file rehab_v9i3e31305_app2.pdf]

Multimedia Appendix 2. Case description

Case description

A case description is developed for the three stroke survivors and their respective clinician, focusing on determinants of VirTele use, indicators of stroke survivor empowerment and data which reflected clinician support of psychological needs.

Case 1

Carolina was a 41-year-old female stroke survivor (7 years since stroke) with severe to moderate UE impairment (stage of arm: 3 in Chedoke McMaster). She was no longer receiving rehabilitation services and was leading an active lifestyle (fitness training, stretching postures for UE). She was very comfortable with computers (accessible at home) and didn't use it frequently (less than once a month). Carolina mentioned that a major advantage of VirTele compared to standard therapy was the capacity to show a real-life demonstration of the activity she improved in or has a difficulty with, to the clinician (through telerehabilitation system). She added that it was easier to show (the improvement) instead of just talking about it, especially given that she had memory deficits (due to stroke). Carolina perceived a meaningful change in the affected arm use, as described:

> "*I put on my sweater, my socks, I do my zipper up and stuff. Yeah it's helping me*."

She played exergames every day during the two-month intervention and spent more than 1055,11 minutes or 18 hours on exergames, including 68 autonomous sessions and 16 supervised exergame sessions. Even if she described one of the five games (Fish Frenzy) as: "*Sort of hard*" she was able to sustain motivation to continue playing. In fact, Carolina reported a problem with the avatar of the game Fish Frenzy, which was difficult to control, as the Fish was going too fast or too slow, on the screen, not following the rhythm of the real movements. Therefore, Carolina hated the game Fish Frenzy which made her "*angry*" and "*upset*". As for effort, Carolina encountered minor technical issues such as loss of password, sound or video cut off which were resolved by the technical research team. Overall, she reported that she felt "*very comfortable*" with the technology and that she got used to it as said: "*it just became a regular routine for me*". Carolina reported that she received positive feedback (competence support) from her entourage regarding the technology which encouraged her to use VirTele and felt the urge to show them (Friends and family) a demonstration of how it works. She said:

“*I mean everybody [friends and family] was happy and everybody spoke well about it and stuff so I was like “ok, sure why not”…they thought it was amazing… I need to show them what I do…it is something that I had to do…”.*

Furthermore, Carolina appreciated the interaction with her clinician (relatedness support) as she said:

“It was awesome. I felt very comfortable with him [the clinician]… it was easy to be around him [the clinician]. He had this way of talking which was calm… and effective”.

Carolina demonstrated empowerment through autonomous decisions and actions (choice of parameters of difficulties, speak about UE use in daily activities), competencies to handle problems (text the clinician when a problem is faced and discuss the solution together) and feeling belongingness (feeling comfortable around the clinician). She also indicated that her clinician respected her decisions (let her speak and choose the difficulty parameters of exergames) and supported her when she needed help (encouragement, demonstrations, answer to questions, resolve problems) and made her feel comfortable through his calm way of talking. She also pointed out : “*I can do stuff with my arm but not as hard as I wanted to. So I motivated myself.*”. Furthermore, she kept using her affected UE at the end of VirTele, in daily activities such as getting dressed, washing hair, etc.

Carolina’s clinician (male) reported during the interview that 2 or 3 telerehabilitation sessions in a week, increased compliance to rehabilitation program, at a frequency (quantity of use of the exergames) that is much more interesting than face-to-face sessions and at a more affordable cost. He said:

“*This is a very good motivational tool. I don't know if this is just the context of the study, but I have never seen patients as motivated as that…using a technology, a game, with a regular monitoring, I think that has a lot to do with it*”

According to him, this contributed not only to the motivation of Carolina but also to her empowerment. He also appreciated the monitoring option of the participant's results (through the exergame platform) and the exergame graphics and color, which he described as :

“ …*much more dynamic and interesting to the eye than other software that may exist in a healthcare context*”.

The clinician also noticed an improvement in the affected arm, mainly in the shoulder area as he explained that there were not many games that were specifically targeting the extension of the elbow and the hand. Regarding exergames, he indicated that the exergame is a good start, but at a certain point the exercises become redundant, as there was not a lot of variability in the levels of difficulty and progression. He suggested that it might be advantageous to use different shapes or different directions, other than what already exists. He also reported that the participant always forgot to recalibrate (Kinect camera detection of movements) before she started playing which explains why she had difficulties with Fish Frenzy. The clinician reported having a lot of apprehension regarding the lack of “*physical contact*” to simulate and demonstrate the movement, at the start of the intervention, as he said: “*I had the impression that this was not optimal just by telecommunication*.”. The clinician’s apprehension decreased during the intervention, as Carolina was able to understand the instructions and demonstrations.

As for effort, the clinician found the platform intuitive and easy to use both from his side or from the participant side. He mentions the same technical issues as those mentioned by Carolina, which he managed to resolve technically (Call the participant and give instructions on where to click) and emotionally (calm the participant down, explain that it’s not her fault), over time. Finally, the clinician reported that the safety of the participant and the establishment of a trusting relationship with the latter facilitated his experience with VirTele, even if he had a lot of apprehension prior to starting the intervention. Furthermore, the clinician corroborated Carolina's statements on the support of her autonomy (let the participant choose the level of difficulty and games) and adds that he felt trusted by Carolina, corroborating the support of relatedness.

The clinician’s logbook indicated that self-directed exercises (in addition to exergames) were performed by Carolina, during the VirTele intervention, such as elbow flexion and extension using a stick, writing with the affected UE and shoulder abduction and adduction using a stick.

The clinician’s logbook indicated also that he used BCT’s techniques and motivational techniques such as reflective listening (express empathy) (Table 2), which support competence, relatedness and autonomy.

Case 2

Helene was an 89-year-old female stroke survivor (7 years since stroke) with moderate UE impairment (stage of arm: 5 in Chedoke McMaster). She was no longer receiving rehabilitation services and was doing some exercises for her affected UE. She was not comfortable with computers and had never used one before. Helene indicated that she had no expectation regarding the outcomes of the intervention on her affected UE and was negative regarding the intervention. However, when she received the technology at home, she said:

"*I conditioned myself to do it [exergames] at least two or three times a day. I really liked the experience.*"

When compared to standard therapy, Helene described VirTele, as follows:

"*I find your system much more elaborate ... Maybe because I had other exercises before. But your system, I find it great for helping people exercise. I was doing them less and less[the standard exercises].*"

Helene spent 1178,32 minutes or 20 hours on exergames and used them at least 5 times a week, including 37 autonomous sessions and 12 supervised exergame sessions. She stated that she perceived no important change on her UE since the beginning of the experience, but she was able to sustain motivation to continue playing, for external reasons such winning. In fact, Helene enjoyed the playful aspect of exergames which she compared to "*Bridge card games*" and said:

" *I'm a winner! Young, I was a winner! It had to work!...Not always winning, but you have to make it work.*"

However, she pointed out a problem with the avatar of the Kitchen clean up game, which didn't follow the real hands movements. As for effort, Helene reported that she had difficulty launching the video-conferencing system and that it was her daughter who helped her turn on the system. On the other hand, she managed to start up the exergames by herself.

Helene indicated that her daughter not only helped her use the technology but also encouraged her to start and continue using VirTele. Helene also pointed out how positive, encouraging and motivating her clinician was during the video-conference sessions. In addition, Helene reported that she felt comfortable interacting with the clinician who was patient and enthusiastic and

supported her to use exergames and UE in activities of daily life, through advices on performance (performance of UE during exergames and some activities).

After the end of intervention period, Helene felt more motivated to resume physical activities to avoid the deterioration of her health condition (loss of autonomy, stiffening of the arm, chronic deficits of arm due to stroke) and use her UE in daily activities such as making her bed, combing her hair.

In an interview with Helene's clinician (female), she reported that the VirTele technology was very good for reducing travel, especially for people who have limited access to rehabilitation services and given that face-to-face intervention is no longer necessary at this stage (7 years post stroke). Prior to starting VirTele, the clinician apprehended the lack of the "*hands on*" to demonstrate a movement or show a compensation as she explained:

"*when we are face to face, it is easier because we are able to touch with our hands, we are able to demonstrate with our hands, but this [VirTele] is more like a different approach*"

The clinician's apprehension decreased over time as the stroke survivor was able to understand the instructions provided on how to perform the exergame and use their UE. The clinician also noticed a high compliance of Helene to exergames and a more frequent use of UE in activities of daily life as described by the participant. As for effort, the clinician reported only minor technical issues (video cut off and internet problem) which were managed by Helene's daughter or the clinician. She added that the use of the technology by Helene who had no previous experience with computers, was very difficult. According to the clinician, it is important to be at least comfortable at turning on a computer and using a mouse. The clinician stated that it was safe to provide the intervention at home since it implies performing exercises while sitting (no risk of falling) and pointed out that the videoconference component and shared decision making component played an important role in establishing a trusting relationship with the participant. Furthermore, the clinician indicated that she included Helene in every decision made:

"*I make sure to share my thought and see why I am progressing, why I am not increasing the difficulty; then I also take their [Helene and Jack] opinion, then I try to compromise*"

The clinician's logbook indicated that Helene often felt fatigued after exergames sessions, and that no complementary exercises were suggested. The logbook' notes also indicated that the

clinician used BCT's and motivational techniques (eg, reflective listening) (Table 2), which support competence, relatedness and autonomy.

Case 3

Jack was a 50-year-old male stroke survivor (4 years since stroke) with moderate impairment of the UE (stage of arm: 4 in Chedoke McMaster). He was no longer receiving rehabilitation services and was still using his UE for some household activities. He was very comfortable with computers (accessible at home) and used it at least once a week. He was the only participant with previous experience in information technology and videogames (he used to work as engineer in a company of videogames). Prior to starting the intervention, Jack had some hesitation and questions about the therapeutic value of exergames, which evolved over time with practice and resolved, as he said:

> "*There were question marks which were quickly resolved by doing them [exergames]. There have been cases where I said to myself: um! Will it really do me good? After five times of practice, I saw the things that I understood was useful. It became more interesting*."

Jack appreciated the exergames as he said: " *it was a good activity to do because it's short, it's straightforward, you knew exactly what to do*". He spent 996,97minutes or 17 hours on exergames and used them at least 5 times a week, including 46 autonomous sessions and 12 supervised exergame sessions. Jack reported that he felt better and described the change in his affected UE as subtle and positive. Jack believed that VirTele could be useful at the end of in-patient and outpatient rehabilitation services, depending on the stroke survivor's level. He also suggested that exercises could be taught while in clinic:

> "*…people have things and exercises to learn before. Then, when they are ready enough, we offer them the exercises [VirTele]*."

Jack pointed out to one limit of exergames which was the lack of sufficient rest time between sets of repetitions in each game and suggested a break of 10 or 20 seconds. As for effort, Jack indicated that it was easy to use the system due to his previous experience and that he faced two major problems, the first related to internet access and the second related to his difficulty to communicate his ideas with the clinician due to aphasia. He also added that he could place the technology material (computer and Kinect camera) in his office.

Jack demonstrated autonomy in making decisions related to choices of exergames and level of difficulty, and indicated that the clinician supported his use of exergames, through tips, demonstrations and feedback. He also pointed out that he was autonomously motivated to play exergames and no one forced him to do it. Jack, who was followed by the same clinician as Helene, indicated that she was kind.

In addition to what was reported in case two, Jack's clinician indicated that a ceiling effect was rapidly reached in the difficulty level of exergames, for this participant, which impacted his motivation. She added that Jack understood the instructions and that it was easy to work with him. Minor technical issues were identified (the screen froze or slowed down, limited access to internet, faced by Jack). The clinician pointed out communication challenges faced during videoconference sessions with Jack due to the aphasia, which made it difficult for her to understand Jack's needs and customize the intervention appropriately.

The clinician also stated that the participant had demonstrated a lot of resistance to using his affected UE in daily activities, even if he was responsible and compliant, with regards to exergames.

Finally, the clinician indicated that she supported Jack's autonomy through shared decision making and Jack's relatedness through reflective listening. Regarding competence support, Jack's clinician indicated that she managed to show the participant that he was able to succeed in the exergames by valorizing small successes and encouraging him to maintain some positions, even for a few seconds.

The clinician's logbook indicated that self-directed exercises (in addition to exergames) were performed by Jack, during the VirTele intervention period and included, for example, elbow extension and supination, use of UE to turn on the lights, shoulder abduction and adduction using a stick. Furthermore, the logbook notes indicated that the clinician used BCT's and motivational techniques (eg, reflective listening) (Table 2), which support competence, relatedness and autonomy.
