# Supplementary material for: Lessons Learned From Clinicians and Stroke Survivors About Using Telerehabilitation Combined With Exergames: Multiple Case Study
Source: JMIR Rehabil Assist Technol. 2022 Sep 15;9(3):e31305. doi: 10.2196/31305 (PMC9523527; doi:10.2196/31305)
Supplement: Multimedia Appendix 3 [file rehab_v9i3e31305_app3.pdf]

Multimedia Appendix 3. Indicators of empowerment and support of psychological needs among the three cases

<table>
<tr><th></th><th>Support of psychological needs by the clinician</th><th>Empowerment of the stroke survivor</th><th>consequences</th></tr>
<tr><td>Autonomy</td><td>-Give the opportunity to the participant to speak about UE use in daily activities and encountered difficulties
-Accept to modify the parameters of difficulty of exergames according to the participant preferences.
-9.2 allow participant express advantages and disadvantages
-Shared decision making
-9.2 allow participant express advantages and disadvantages,
-Shared decision making
-9.2 allow participant express advantages and disadvantages</td><td>- Speak about UE use in daily activities
-Choose the parameters of difficulty of exergames(“Make it faster, make it slower”)
-taking decisions related to choice of exergames and level of difficulty</td><td rowspan="2">- High adherence to exergames
- use affected UE in daily activities, maintained after the end of VirTele
-High adherence to exergames
-Play exergame for enjoyment
-Resume physical activities to avoid the deterioration of health condition
-use affected UE in daily activities, maintained after the end of VirTele
-High adherence to exergames
- Resistance to use the affected UE in daily activities</td></tr>
<tr><td>Competence</td><td>-Answer to the participant questions and help them resolve the problem faced.
-encourage and show the participant how to stretches and exercise with affected arm
-demonstration of exercises
- 15.1verbal persuasion about capability
-1.1goal setting
-1.5goal review
-1.4action planning
- 1.2problem resolving
-2.7 feedback on behavior results (positive feedback)
-15.1verbal persuasion about capability,
-1.1goal setting,
-1.5goal review
-1.4action planning
-1,2problem resolving
-2.2 feedback on behavior
-2.7 feedback on behavior results
-7.1 instructions and indices</td><td>-“if I had a problem or a question, I text him “
-Feeling supported to do exercises and stretches of the arm through demonstration and encouragement.
- Feeling supported to play exergames and use UE in activities of daily life through advices on performance
- Feeling supported to use exergames through advices, demonstrations and feedback.</td></tr>
</table>

	--advices on performance during exergames<br>-Support the use of exergames through advices, demonstrations and feedback.<br>-Rewarding small successes and encouraging to maintain some postures, even for a few seconds.<br>-15.1verbal persuasion about capability<br>- 1.1goal setting<br>- 1.5goal review<br>-1.4action planning<br>-1,2problem resolving<br>-2.2 feedback on behavior<br>-2.7 feedback on behavior results<br>-7.1 instructions and indices		
**Relatedness**	-Have a calm way of speaking.<br>-establishment of a trusting relationship<br>-reflective listening (express empathy)<br>- Reflective listening (express empathy)<br>-listen and acknowledge the participant' opinion<br>- be patient and enthusiastic<br>-listen and acknowledge the participant' opinion<br>-reflective listening(express empathy)	-Feeling comfortable and easy to be around and work with the clinician.<br>- feeling comfortable interacting with the clinician<br>-Find the clinician kind	

Case 1= in blue; Case 2= in orange; Case 3= in purple.
