# Supplementary material for: Lessons Learned From Clinicians and Stroke Survivors About Using Telerehabilitation Combined With Exergames: Multiple Case Study
Source: JMIR Rehabil Assist Technol. 2022 Sep 15;9(3):e31305. doi: 10.2196/31305 (PMC9523527; doi:10.2196/31305)
Supplement: Multimedia Appendix 4 [file rehab_v9i3e31305_app4.pdf]

Multimedia Appendix 4. Clinicians’ recommendations regarding using telerehabilitation combined with exergames

- This technology targets survivors who still have some functionality in the forearm and hand.
- Aphasia does not affect the technology use, as long as the survivor is able to understand the clinician's instructions. However, it is important to note that it limits the interaction with the clinician (ideas exchanges, discussion, motivational interviewing) and may cause frustration on the part of the survivor.
- Survivors with limited computer skills who can be assisted by a caregiver in their use of the technology, are eligible for this type of intervention.
- Clinicians are encouraged to demonstrate the exercises to participants to ensure they are well understood.
- Clinicians’ instructions have to be clear, simple, and concise when showing a movement or an exercise. The movements may be showed from different angles and the participant may be asked to repeat the exercise, to ensure the correct reproduction
- Attention should be paid to the quality of movement during an exercise. For example, the clinician may ask the participant to concentrate or be more aware of the movements of the affected arm (eg, maintain supination of the arm, keep the shoulder elevated, etc.).
- It is important to support small successes, for example maintaining supination for few seconds, succeed in an exercise or correctly achieve an activity.
- In survivors with severe impairment, attention should be paid to the quality of movements (movements outside the pathological schema) and not quantity, which implies a slow progression in the exergames’ level of difficulty.
- Progression in difficulty level of the exergames, may be based on the achievement of the movement quality, the absence of pain and the participant's perception (finds the exercise easy).
- The exergames’ scores may not always be used as a benchmark measure to make a decision related to the difficulty level. However, they can be a source of motivation for survivors to persevere during the game.
- In addition to the exergames, activities involving the use of the affected hand (cut vegetables, make the bed, etc.) may be added to the rehabilitation program, since the hand is not targeted by the Kinect during exergames.
- Weekly objectives may be established in partnership with the participant, related to exergames adherence (frequency of use, duration, etc.) and compliance to activities of daily life, for example cut vegetables (size or quantity of vegetables, frequency, etc.).

- It is important to remind the participant, the link between the movements involved in the exergames and their relevance to the successful accomplishment of activities of daily life.

- Shared decision making (goal setting, choice of games and activities, discuss the difficulty level, etc.) during video conferences enhance the participant’s trust in the therapist, which is difficult to establish, using only asynchronous technology.

- The role of the teleclinician in adopting this approach is to motivate, explain, reassure, and demonstrate the exercises, in order to help survivors attain their objectives.

- To help survivors become familiar with the technology, two to three training sessions are required, including a remote practice session (similar to an in-person the session with the clinician). The period between training and the first telerehabilitation session, should be short (less than one week) to avoid memory loss.

- A brochure showing the different interfaces of the technology with simple instructions is suggested to accompany the use of the technology and serve as a reminder.

- Intermittent face-to-face sessions (every two weeks) are suggested to collect objective data of motor function (eg, administer the Fugl Meyer), to do physical examination (movement quality) or interventions such as manual therapy (to address range of motion when there is no progression).
